# Pak1ip1 Loss-of-Function Leads to Cell Cycle Arrest, Loss of Neural Crest Cells, and Craniofacial Abnormalities

**DOI:** 10.3389/fcell.2020.510063

**Published:** 2020-09-01

**Authors:** Alexios A. Panoutsopoulos, Angelo Harlan De Crescenzo, Albert Lee, Amelia MacKenzie Lu, Adam P. Ross, Laura N. Borodinsky, Ralph Marcucio, Paul A. Trainor, Konstantinos S. Zarbalis

**Affiliations:** ^1^Department of Pathology and Laboratory Medicine, School of Medicine, University of California, Davis, Davis, CA, United States; ^2^Institute for Pediatric Regenerative Medicine, Shriners Hospitals for Children – Northern California, Sacramento, CA, United States; ^3^David B. Falk College of Sport and Human Dynamics – Department of Public Health, Syracuse University, Syracuse, NY, United States; ^4^Department of Physiology and Membrane Biology, School of Medicine, University of California, Davis, Davis, CA, United States; ^5^Department of Orthopedic Surgery, School of Medicine, University of California, San Francisco, San Francisco, CA, United States; ^6^Stowers Institute for Medical Research, Kansas City, MO, United States; ^7^Department of Anatomy and Cell Biology, School of Medicine, The University of Kansas Medical Center, Kansas, KS, United States; ^8^MIND Institute, University of California, Davis, Davis, CA, United States

**Keywords:** neural crest, development, orofacial clefts, mouse, Pak1ip1, ribosomopathies

## Abstract

Neural crest cells (NCCs) comprise a transient progenitor cell population of neuroepithelial origin that contributes to a variety of cell types throughout vertebrate embryos including most mesenchymal cells of the cranial and facial structures. Consequently, abnormal NCC development underlies a variety of craniofacial defects including orofacial clefts, which constitute some of the most common birth defects. We previously reported the generation of *manta ray* (*mray*) mice that carry a loss-of-function allele of the gene encoding the preribosomal factor Pak1ip1. Here we describe cranioskeletal abnormalities in homozygous *mray* mutants that arise from a loss of NCCs after their specification. Our results show that the localized loss of cranial NCCs in the developing frontonasal prominences is caused by cell cycle arrest and cell death. In addition, and consistent with deficits in ribosome biosynthesis, homozygous *mray* mutants display decreased protein biosynthesis, further linking Pak1ip1 to a role in ribosome biogenesis.

## Introduction

Vertebrate craniofacial morphogenesis is crucially dependent on proper specification, migration, and differentiation of neural crest cells (NCC), a transient population of cells born at the interface of neural and non-neural ectoderm during neurulation (Helms and Schneider, 2003; Minoux and Rijli, 2010). After initial specification, NCC form via epithelial to mesenchymal transition (EMT), delaminate from the developing neuroepithelium, and migrate, long distances, to populate the facial prominences and pharyngeal arches where they give rise to the majority of the bone and cartilage components of the craniofacial skeleton. Defective NCC development may contribute to a range of craniofacial abnormalities (neurocristopathies), including some of the most common, such as orofacial clefts of the lip and/or palate (Trainor, 2010; Cordero et al., 2011). Consequently, understanding the mechanisms governing NCC specification, migration, and differentiation, not only has the capacity to illuminate some of the most intricate aspects of vertebrate development, but also to provide new avenues toward tackling some of the most common congenital anomalies associated with craniofacial dysmorphologies.

Intriguingly, a growing body of research suggests an overlap between neurocristopathies and ribosomopathies, disorders precipitated by impaired ribosome biogenesis, suggesting a particular dependency of NC cells on adequately functioning ribosomes. Ribosome biogenesis is a complex procedure that involves coordination of all three RNA polymerases, ribosomal and accessory proteins as well as more than 70 small nucleolar RNAs (snoRNA) (Nazar, 2004). Disruptions in ribosome biogenesis lead to ribosomopathies with clinical phenotypes that include defects in craniofacial and skeletal development (Trainor and Merrill, 2014). This association has been investigated in considerable detail in Treacher-Collins-Franceschetti syndrome 1, most often caused by mutations in *TCOF1* (Teber et al., 2004), and Diamond-Blackfan anemia, most often caused by mutation in the genes encoding ribosomal proteins S19 and S24 (RPS19, RPS24) (Draptchinskaia et al., 1999; Gazda et al., 2006).

We previously reported a pivotal role of Pak1ip1, a preribosomal factor required for proper 60S ribosomal subunit biosynthesis (Saveanu et al., 2007), in craniofacial development (Ross et al., 2013). Loss of Pak1ip1 in mice leads to midfacial clefting affecting maxillae and secondary palate. *In vitro*, Pak1ip1 has been shown to directly interact with the E3 ubiquitin ligase Mdm2 by which it regulates Tp53-mediated cell cycle progression (Yu et al., 2011). Interestingly, both Pak1ip1 overexpression and inhibition has the capacity to induce Tp53-dependent G1 cell-cycle arrest by either inhibiting Mdm2, leading to Tp53 accumulation, or increasing levels of freely circulating ribosomal proteins L5 and L11 causing nucleolar stress (Yu et al., 2011).

Here, we demonstrate that in mice homozygous for *Pak1ip1* mutation (*Pak1ip1*^*mray/mray*^), cranial NCC survival is impaired leading to malformation of NCC-derived structures. Further, we demonstrate that Pak1ip1 loss *in vivo* leads to G1-cell cycle arrest that predominantly affects the developing frontonasal prominences.

## Materials and Methods

### Ethics Statement

Mice were housed in facilities approved by the Association for Assessment and Accreditation of Laboratory Animal Care International (AALAC). All animals were handled in accordance with protocols approved by the University of California at Davis Institutional Animal Care and Use Committee.

### Animal Husbandry and Genotyping

The colony of animals carrying the *Pak1ip1*^*mray*^ allele (induced on C57BL/6NJ background) is maintained by crossing male carriers with C57BL/6NJ females (from an initial outcross onto FVB/NJ background). This mode of breeding is currently in the sixth generation without any changes in penetrance or variability of the mutant phenotype. All embryos presented in the phenotypic analysis of this study were produced from carriers crossed for at least three generations onto C57BL/6NJ. Routine PCR-based genotyping was performed as previously described (Ross et al., 2013). Embryos of all developmental stages analyzed, were recovered after timed pregnancies and, except for skeletal stainings, fixed by immersion in 4% paraformaldehyde (PFA) in phosphate buffered saline (PBS) (pH 7.4). For each marker and developmental stage shown, we analyzed per experiment at least five embryos of each genotype (WT, MT) and carried out every experiment at least twice.

### X-gal Staining of Whole-Mount Embryos

Embryos were dissected in PBS and fixed for 45 min at room temperature in 4% PFA/PBS. Subsequently, embryos were washed in detergent rinse [0.1 M phosphate buffer (pH 7.3), 2 mM MgCl_2_, 0.01% sodium deoxycholate, and 0.02% Nonidet P-40] and then stained for 48–72 h at room temperature on a rocking platform using 1 mg/ml X-gal in staining solution (detergent rinse with 5 mM potassium ferricyanide and 5 mM potassium ferrocyanide) as a substrate for the detection of β-galactosidase (β-gal) activity. Staining was terminated after visual inspection by repeated washing in PBS and fixation in 4% PFA/PBS until further examined and documented.

### RNA *in situ* Hybridization

All RNA *in situ* hybridization on whole-mount embryos was performed using standard procedures as previously described (Zarbalis and Wurst, 2000). Plasmids to transcribe riboprobes for *Nrp2* and *Ccng1* were previously described.

### Immunofluorescent Analysis

Immunofluorescence was carried out on slide-mounted sections cut at 12 μm from OCT-embedded tissue fixed in 4% PFA/PBS and cryoprotectively frozen in 30% sucrose. Tissue was fixed in ice cold 4% PFA/PBS (pH 7.4) and subjected to antigen retrieval in Decloaking Chamber Pro (Biocare Medical, Pacheco, CA, United States) submerged in DIVA buffer, or citrate buffer (10 mM Na-citrate pH 6.0). After blocking in 10% donkey serum in PBS + 0.1% Tween-20 for 1 h, sections were incubated with primary antibodies for Sox10 (1:50, Santa Cruz, Dallas, TX, United States), pHH3 (1:200, Abcam, Cambridge, MA, United States), or Pax3 (1:200, DHSB, Iowa City, IA, United States). After repeated washes with PBS, tissue was incubated with appropriate secondary antibodies (Alexa Fluor 488 and 596 at 1:200) in 1% BSA in PBS (Alexa Fluor, Invitrogen, Carlsbad, CA, United States). Prior to the final PBS rinse, cells were counterstained with the fluorescent nucleic acid stain 4′,6-diamidino-2-phenylindole (DAPI) (Invitrogen, Carlsbad, CA, United States), coverslipped, and imaged. Numbers of pHH3^+^ and Sox10^+^ cells were counted and ratios of labeled cells over total number of cells (DAPI) within the neural tube of each section established. This approach normalized for the typically smaller size of mutant embryos and revealed significant changes.

### TUNEL Assay

Embryo sections were processed with the DeadEnd Fluorometric TUNEL System (Promega, Madison, WI, United States) according to manufacturer’s instructions. First, sections were fixed in 4% PFA, then permeabilized with 4 μg/ml solution of Proteinase K. Afterwards, sections were equilibrated with the proprietary equilibration buffer, then labeled with a nick-end-specific fluorophore, counterstained with DAPI, coverslipped, and confocally imaged. Numbers of TUNEL^+^ cells were counted and ratios of labeled cells over total number of cells (DAPI) within the neural tube of each section was established.

### Skeletal Staining

After collection, embryos of varying developmental stages were skinned and internal organs removed. Subsequently, embryos were transferred to 95% ethanol for 24 h and acetone for another 24 h. Next, the embryos were stained in a solution containing a mix of alcian blue and alizarin red dyes (85% ethanol, 5% glacial acetic acid, 5% of 0.3% alcian blue in 75% ethanol, and 5% of alizarin red in 95% ethanol) for 24 h. After discarding staining solution, the embryos were washed with 70% ethanol for at least 24 h and then transferred to 1% potassium hydroxide solution for another day. After initial clearing, embryos were further macerated in 20% glycerol/80% of 1% potassium hydroxide for multiple days (depending on size) until desired clearing was achieved. For storage and subsequent documentation, embryos were transferred to a 1:1 glycerol/ethanol solution at room temperature.

### O-Propargyl-Puromycin (OPP) Protein Synthesis Assay

Embryos were dissected out of the uterus and placed in a 1:2000 solution of O-propargyl-puromycin (OPP) reagent (Click-iT Plus OPP Alexa Fluor 488, Invitrogen, Carlsbad, CA, United States) in DMEM and left to incubate at 37°C for 30 min. Following this incubation, yolk sacs and amnions were removed to be processed for genotyping while embryos were fixed in 4% PFA for 30 min. After fixation, embryos were stored at 4°C in PBS containing 0.01% sodium azide. Click-it reaction was performed according to the manufacturer’s instructions followed by tissue permeabilization with 1% Triton X-100 in PBS and subsequent 5 min incubation in a 1:50 solution of TrueBlack Lipofuscin Autofluorescence Quencher (Biotium, 23007-F) to diminish autofluorescence. All embryos were imaged at the same magnification (77×), laser power, and exposure time and images were analyzed using ImageJ software to assess fluorescence intensity by measuring the integrated density of the embryo, and subtracting the integrated density of the background. Negative controls to detect and potentially normalize to autofluorescence did either (i) not include OPP reagent during incubation or (ii) omitted click-it reaction. Neither control produced any discernable signal at the illumination and exposure conditions that we used to document outcomes. Thus, OPP outcomes were quantified and directly compared between genotypes. Statistics were performed on GraphPad Prism version 8 using unpaired *t*-test.

### Embryo Imaging and Histology

Whole-mount imaging was performed on a Zeiss Lumar.V12 stereo-microscope (Oberkochen, Germany) with attached Zeiss AxioCam MRc camera and associated AxioVision software (version 4.8.2). Fluorescent imaging of tissue sections was performed on a Nikon A1 scanning confocal laser microscope with associated software (Nikon Instruments, Tokyo, Japan).

### Cell Cycle Analysis via Propidium Iodide Incorporation and Flow Cytometry

Embryos of stages E10.5, E12.5, and E14.5, were harvested, heads removed, and equivalent amounts minced with a fine razor blade. Minced tissues were suspended in Hank’s Balanced Salt Solution (HBSS) without calcium and magnesium, centrifuged at 300 × *g* at 4°C for 5 min, and incubated in 0.025% Trypsin/EDTA at 4°C for 2 h. Subsequently, dissociated tissues were strained through a cell strainer by rinsing with HBSS and samples run through Life Technologies Attune NxT Flow Cytometer to determine our live population. Samples passing a threshold of 1 × 10^6^ live cells were then fixed by adding 500 μl of 100% methanol. After centrifugation at 300 × *g* at 4°C for 10 min, supernatants were discarded and samples were ran again through the Flow Cytometer to determine numbers of the fixed population. Lastly, cells were resuspended in 500 μl of propidium iodide/RNase staining solution (Cell Signaling Technologies, Danvers, MA, United States) before running them again through Life Technologies Attune NxT Flow Cytometer. FlowJo was used for gating and analysis. GraphPad Prism (version 7) software was used to perform ANOVA to calculate statistical significance (GraphPad Software, La Jolla, CA, United States).

### Reverse Transcription Quantitative PCR

Whole E10.5 embryos were homogenized on ice and total RNA was extracted by using the Quick RNA miniprep kit (Zymo Research, Irvine, CA, United States) following the manufacturers’ directions. Subsequently, cDNA was generated from 5 μg of total RNA using Omniscript RT kits (Qiagen) following the manufacturers’ instructions. Real time qRT-PCR was conducted in a Stratagene Mx3005P QPCR Systems machine (Agilent Technologies, Santa Clara, CA, United States), at 55°C annealing temperature, 40 cycles, and using the following primers: for *Ccng1* RT forward: 5′- tcatctgacttcccagatttgat-3′, *Ccng1* RT reverse: 5′- tgcattcatgaaatgttggttt-3′; control *Actb* RT forward: 5′- gatcattgctcctcctgagc-3′, control *Actb* RT reverse: 5′- agtccgcctagaagcacttg-3′.

## Results

### Homozygous *mray* Mutants Display NC Cell Loss Due to Cell Cycle Arrest

**FIGURE 1:**
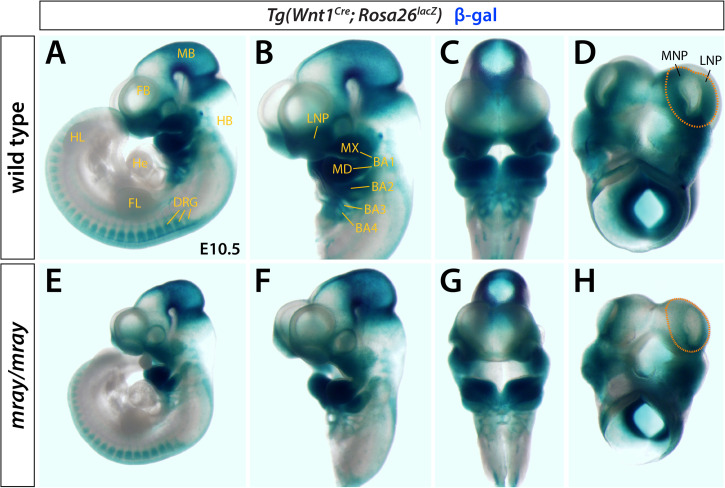
*Pak1ip1*^*mray/mray*^ embryos display NCC loss. X-gal whole-mount staining indicative of β-gal activity reveals distribution of Wnt1^+^ cells in *Pak1ip1*^*mray/mray*^; *Wnt1*^*Cre*^; *Rosa26*^*lacZ*^ E10.5 embryos compared to WT control. Lateral **(A,B,E,F)** and frontal **(C,G)** views of the embryos, as well as ventral views of the heads only **(D,H)** demonstrate reduced expression in the frontonasal prominences of the mutant. The domains of greatest signal reduction in *mray/mray* are outlined in WT **(D)** and mutant **(H)** (orange dashed line). NC migration is not affected in most structures of mutant embryos, such as dorsal root ganglia (DRG) and mandibular prominences (MD). In midbrain (MB), a moderate increase in signal intensity can be observed in the mutant. BA1-4: branchial arches 1-4, FB: forebrain, FL: forelimb, FNP: frontonasal prominence, HB: hindbrain, He: heart, HL: hindlimb, LNP: lateral nasal prominence, MB: midbrain, MNP: medial nasal prominence, MX: maxillary prominence.

Homozygous *mray* mutants (*Pak1ip1*^*mray/mray*^) exhibit orofacial midline clefting, possibly caused by a failure of midfacial outgrowth and/or inability of the maxillary processes to fuse along the midline. To examine whether NCC formation, migration, and/or differentiation contribute to this craniofacial defect, we chose a genetic approach by crossing *Wnt1-Cre*; *R26R-lacZ* reporter mice (Danielian et al., 1998) into the background of *mray* mutant mice. This reporter indelibly labels NCC and their cell and tissue derivatives throughout development. We collected embryonic day (E) E10.5 embryos and stained them for β-gal expression, as this stage allows for sufficient NCC migration to identify migration defects and takes into account the delayed development of most *Pak1ip1*^*mray/mray*^ embryos (∼80%). Whole-mount expression analyses revealed a substantial reduction in staining intensity and distribution within the facial primordia of homozygous *mray* mutants (Figure 1). Interestingly, while frontal and lateral nasal processes of the *Pak1ip1*^*mray/mray*^ embryos showed a strong reduction in β-gal^+^ cells compared to control embryos, no other domains appeared diminished in β-gal expression, including branchial arches, and dorsal root ganglia (Figure 1). In contrast, midbrain showed a discernable increase in β-gal expression possibly associated with the fact that it typically shows to be less affected by size decrease compared to other brain areas and to sometimes show even size increase as previously reported (Ross et al., 2013).

**FIGURE 2:**
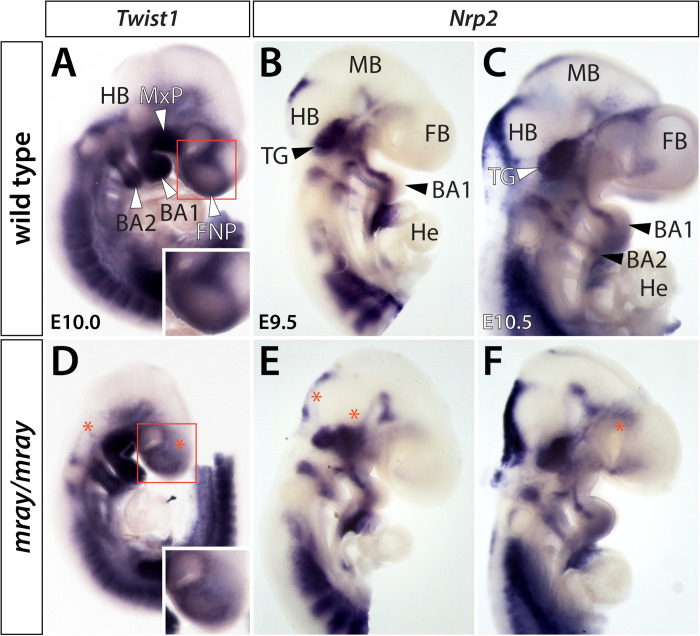
Cranial ganglia are malformed in *Pak1ip1*^*mray/mray*^ embryos. Lateral views of *Twist1* and *Nrp2* expression in WT **(A–C)** and mutant **(D–F)** whole-mount embryos at E9.5–E10.5. **(A,D)** *Twist1* expression labels NCC populated craniofacial and axial structures. Decreased *Twist1* levels can be observed in the frontonasal prominences (FNP) and dorsal to branchial arches (BA) 1 and 2 of the mutant (asterisk). The higher magnification boxed areas in panels **(A,D)** further illustrate decreased *Twist1* expression in the mutant. **(B,C,E,F)** *Nrp2* labels discrete segregated streams of migrating NC cells that colonize the trigeminal ganglia (TG) and branchial arches. In *Pak1ip1*^*mray/mray*^ embryos **(E,F)**, the trigeminal ganglia are misshapen while hindbrain (HB) and forebrain (FB) show additional domains of diminished expression (asterisks). He: heart, MB: midbrain.

To determine whether additional NCC-derived structures may exhibit overt defects, we applied a series of molecular markers by whole-mount RNA *in situ* hybridization. Specifically, we examined expression of *Twist1* at E10.0, as well as *Nrp1* and *Nrp2* at developmental stages ranging from E9.5 to E10.5. Of these, *Twist1*, a regulator of NCC specification (Soo et al., 2002), appeared most informative as it strongly labels aspects of the craniofacial mesenchyme including the medial and lateral nasal processes. Comparing expression levels between genotypes, we noticed overtly reduced *Twist1* expression levels in the facial prominences of the mutant mirroring our observations in *Wnt1-Cre*; *R26R-lacZ* reporter mice and diminished expression in the hindbrain in proximity to branchial arches 1 and 2 (Figures 2A,D). In contrast, *Nrp1* did not show high level expression in the frontonasal mesenchyme and no overt differences between WT and mutant in other domains (data not shown). *Nrp2*, which encodes a guidance factor for cranial NCC (Gammill et al., 2007; Schwarz et al., 2008) revealed subtle morphological changes of the developing cranial ganglia, predominantly of the trigeminal ganglion and variations in hindbrain expression (Figures 2B,C,E,F).

**FIGURE 3:**
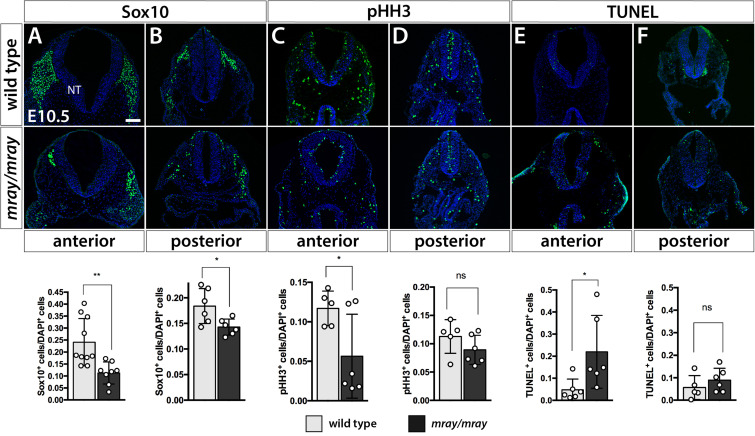
Specific NCC loss in the developing mesencephalic neuroepithelium of *Pak1ip1*^*mray/mray*^ embryos. Immunofluorescent analysis for Sox10 and pHH3, as well as TUNEL assay of the embryonic neural tube (NT) at the level of the midbrain. **(A)** The number of cranial Sox10^+^ migratory NCC is significantly reduced (expressed as a ratio of Sox10^+^ cells over total cells within the neural tube) in anterior/midbrain positions. **(B)** In posterior/truncal positions, a significant, but smaller, reduction in Sox10^+^ cells can be confirmed as well. **(C,D)** Immunofluorescent analysis of pHH3 shows a decrease in the ratio of mitotic cells at the anterior position of the mutant, but no significant effect posteriorly. **(E,F)** TUNEL staining shows a substantial ∼5-fold increase in the ratio of apoptotic cells within the anterior neuroepithelium of the mutant, but no significant differences between genotypes at truncal positions. Scale bar is 100 μm. *p < 0.05 and **p < 0.01.

Our findings of reduced cranial NCC-specific labeling in the facial prominences of *Pak1ip1*^*mray/mray*^ embryos prompted us to examine whether cranial NCC are specifically affected compared to NCC of other regions. Immunofluorescent analysis at E10.5 of Sox10^+^ migrating NCC of the mesencephalic region that contribute to the frontonasal prominences revealed a significant 2.5-fold decrease in their numbers in *Pak1ip1*^*mray/mray*^ compared to WT embryos (WT 0.2409 ± 0.03132, mutant 0.1127 ± 0.01635, *p* = 0.0039; Figure 3A). A lesser, although still statistically significant, decrease (∼20%) was detected posteriorly for the truncal NC at the level of the hindlimb (WT 0.1839 ± 0.01395, mutant 0.1429 ± 0.006427, *p* = 0.0236; Figure 3B). These changes were unrelated to any specification defects within the neural tube as Pax3 immunostaining revealed proper dorsoventral patterning of the neuroepithelium in affected *mray* mutants (Supplementary Figure S1). Considering Pak1ip1’s role in ribosome biogenesis, which if disrupted may lead to cell cycle arrest and cell death, we deiced to examine the contribution of proliferation and apoptosis to the loss of Sox10^+^ cells in the developing mesencephalic neuroepithelium. Indeed, evaluating proliferative rates by the mitotic marker pHH3, we identified a significant ∼60% decrease in the percentage of pHH3^+^ cells in the anterior neural tube of homozygous mutants compared to controls (WT 0.1171 ± 0.009794, mutant 0.05648 ± 0.02169, *p* = 0.0419; Figure 3C), while no statistically significant differences were detected posteriorly (WT 0.1128 ± 0.01325, mutant 0.08926 ± 0.01049, *p* = 0.1917; Figure 3D). Assessing the ratio of apoptotic neuroepithelial cells by TUNEL assay, we discovered a significantly five-fold increased rate in *Pak1ip1*^*mray/mray*^ embryos compared to controls in the ratio between TUNEL^+^ to DAPI^+^ cells in the anterior neural tube (WT 0.04771 ± 0.01988, mutant 0.2198 ± 0.06736, *p* = 0.0342; Figure 3E), while no statistical significance was found in the posterior truncal position (WT: 0.05622 ± 0.02396, mutant 0.08963 ± 0.02145, *p* = 0.3250; Figure 3F).

### Cell Cycle Progression Is Arrested and Protein Biosynthesis Reduced in *Pak1ip1*^mray/mray^ Embryos

**FIGURE 4:**
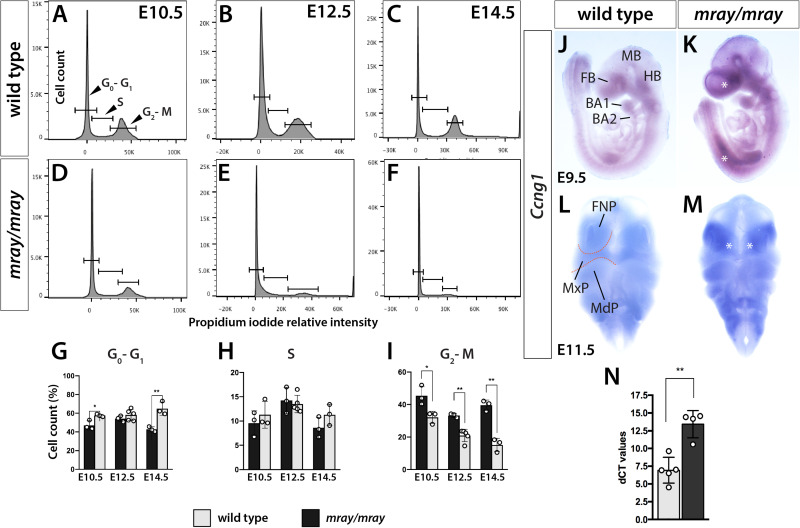
Cell cycle progression is arrested in *Pak1ip1*^*mray/mray*^ embryos. Representative histograms displaying cell cycle profiles of WT control and *Pak1ip1*^*mray/mray*^ embryos obtained by flow cytometry of propidium iodide labeled cells. Three different stages were analyzed, E10.5 **(A,D,G)**, E12.5 **(B,E,H)**, and E14.5 **(C,F,I)** to obtain a stage-dependent developmental delineation of cell cycle progression. **(G)** At all stages, a significant decrease in the proportion of cells in G_2_/M phase can be observed in *Pak1ip1*^*mray/mray*^ embryos with this fraction decreasing as development proceeds. In contrast, the proportion of cells in G_0_/G_1_ stage are significantly increased at E10.5 and E14.5 with this increase being highest at E14.5. *p < 0.05 and **p < 0.01. **(J,K)** Lateral views of *Ccng1* RNA *in situ* hybridization analysis at E9.5 demonstrates upregulated expression in *Pak1ip1*^*mray/mray*^ embryos compared to WT with greatest differences observed at the ventral forebrain (FB) and along the body axis (asterisks). **(L,M)** Frontal views of E11.5 embryos confirm *Ccng1* upregulation in the mutant with greatest increase observed in the frontonasal prominences (FNP) (asterisks). **(N)** The results of real time qRT-PCR confirm a significant upregulation of *Ccng1* transcripts in E10.5 *Pak1ip1*^*mray/mray*^ embryos. BA: branchial arch, FB: forebrain, HB: hindbrain, MB: midbrain, MdP: mandibular process, MxP: maxillary process.

Since our analysis of *Pak1ip1*^*mray/mray*^ embryos indicated a specific loss of cranial NCC, likely mediated by decreased proliferation and increased apoptosis of neuroepithelial cells, we decided to further test the dynamics of cell cycle progression, which is intimately linked to either process, as cell cycle arrest of progenitor cells will lead to decreased proliferative rates and possibly apoptosis. To that effect, we quantified the relative distribution of cells across the cell cycle by detecting DNA content in individualized cells of the heads through flow cytometry at three developmental stages (E10.5, E12.5, E14.5). The method is based on the concept that DNA content, as measured by propidium iodide intercalation and associated fluorescent intensity, increases as proliferative cells progress through the cell cycle from G_0_/G_1_ to S and G_2_/M phase. Testing and comparing the results of the flow cytometric analysis revealed differences between WT and *Pak1ip1*^*mray/mray*^ embryos. Specifically, across all stages examined, the percentage of cells in G_0_/G_1_ phase was increased in homozygous *mray* embryos compared to WT, consistent with an expectation of increased G_1_ cell cycle arrest (E10.5: WT 47.03 ± 2.87, mutant 57.53 ± 2.99, *p* = 0.02; E12.5: WT 54.17 ± 1.85, mutant 58.22 ± 3.80, n.s.; E14.5: WT 42.73 ± 1.58, mutant 64.83 ± 4.45, *p* = 0.007; Figures 4A–I). Furthermore, by comparing the different developmental stages of *Pak1ip1*^*mray/mray*^ embryos we noticed a significant increase in the proportion of cells in G_0_/G_1_ phase as development proceeds, confirming a progressive accumulation of cells in cell cycle arrest (Figures 4A–E). To spatially resolve cells and tissues that are affected by cell cycle arrest, we opted to analyze *Ccng1* distribution. This choice was informed by the fact that ribosome biogenesis defects can lead to Tp53-mediated cell cycle arrest and we had earlier demonstrated Tp53 upregulation in *Pak1ip1*^*mray/mray*^ embryos (Ross et al., 2013). *Ccng1* is a Tp53-responsive gene the activation of which will lead to cell cycle arrest at the G_1_ phase and thus, can serve as a useful marker of mammalian cells inhibited in growth and proliferation (Okamoto and Beach, 1994; Zhao et al., 2003). Indeed, *Ccng1* whole-mount RNA *in situ* hybridization revealed low-level expression throughout WT embryos, but compared to WT visibly upregulated transcript levels in *Pak1ip1*^*mray/mray*^ embryos (Figures 4J–M). Specifically, at E9.5 we detected widespread *Ccng1* upregulation along the body axis of mutant embryos with highest levels observed at the ventral forebrain (Figures 4J,K). At E11.5 and compared to WT, highest *Ccng1* expression in *Pak1ip1*^*mray/mray*^ embryos was observed in all aspects of the developing frontonasal prominences (Figures 4L,M). We sought to further quantify this effect by performing *Ccng1* real time qRT-PCR in lysates of E10.5 embryos. After normalization using *Actb* expression as control, we identified a significant two-fold increase in *Ccng1* transcripts of *Pak1ip1*^*mray/mray*^ embryos compared to WT (Ct values after normalization, WT: 6.922 ± 0.8117, mutant: 13.45 ± 0.955, *p* = 0.0012; Figure 4N). Additionally, performing whole-mount fluorescent labeling with LysoTracker to detect apoptotic cells (Fogel et al., 2012), in later stage embryos (E13.5, E14.5) revealed a substantial increase in apoptotic cells in *Pak1ip1*^*mray/mray*^ embryos compared to WT (Supplementary Figure S2) further confirming the deleterious effect of *Pak1ip1* mutation on cell survival.

**FIGURE 5:**
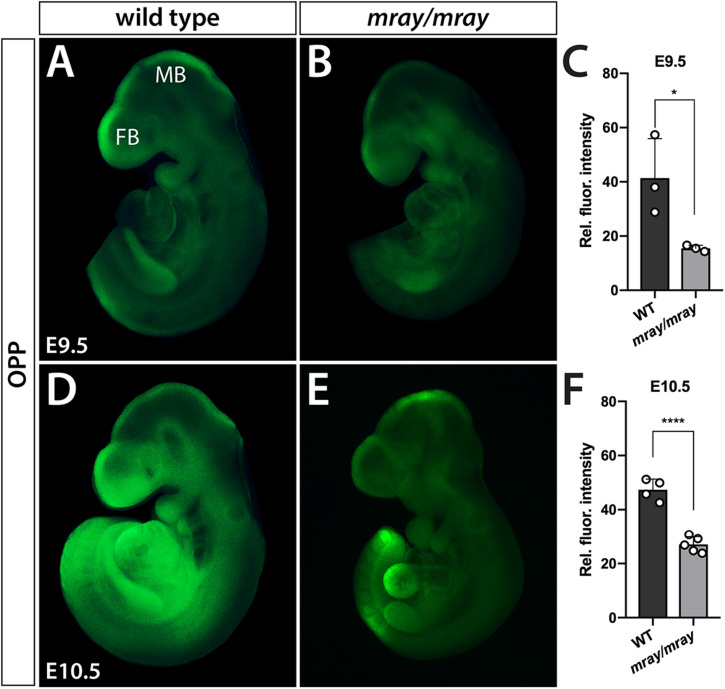
Protein synthesis is reduced in *Pak1ip1*^*mray/mray*^ embryos. Fluorescent analysis of whole-mount embryos incubated with O-propargyl-puromycin (OPP) to evaluate protein synthesis during a 30 min interval reveals significant differences between genotypes. Two developmental stages were assessed, E9.5 **(A–C)** and E10.5 **(D–F)**. Results of fluorescent signal quantification demonstrate either stage reduced levels of relative fluorescence in *Pak1ip1*^*mray/mray*^ embryos compared to WT. *p < 0.05 and ****p < 0.0001.

Considering that Pak1ip1 has been shown to be required for effective ribosome biosynthesis on which protein translation depends, it stands to reason that *Pak1ip1*^*mray/mray*^ embryos may exhibit overt deficits in the rate at which they synthesize proteins. In order to assess overall protein synthesis, we performed OPP protein synthesis assays (Liu et al., 2012). The method is based on the efficient incorporation of OPP, an alkyne analog of puromycin, into newly translated proteins, which upon Click-iT reaction produces a fluorescent signal proportional to the amount of protein synthesis. The results indicated significantly decreased overall protein synthesis in homozygous *mray* embryos at E9.5 and E10.5 compared to controls (E9.5: WT 41.39 ± 8.41, mutant 15.46 ± 8.44, *p* = 0.004; E10.5: WT 47.36 ± 1.43, mutant 27.07 ± 2.27, *p* < 0.0001; Figure 5).

### Deficits in Formation of the Craniofacial Skeleton

**FIGURE 6:**
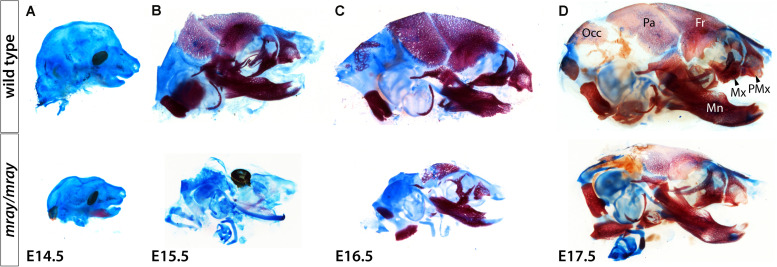
Delayed formation of the craniofacial skeleton in *Pak1ip1*^*mray/mray*^ embryos. Lateral views of skulls stained with alcian blue and alizarin red at different stages confirm delayed skull ossification in *Pak1ip1*^*mray/mray*^ embryos compared to WT. **(A)** Little ossification can be observed at E14.5 irrespective of genotypeA. At E15.5 **(B)** and E16.5 **(C)**, while most bones of the WT skull have formed, in the mutant a distinct delay in ossification can be observed. **(D)** At E17.5, *Pak1ip1*^*mray/mray*^ embryos show most cranial bones formed irrespective of whether they are predominantly of NC or mesodermal origin. Fr: frontal bone, Mn: mandibular bone, Mx: maxillary bone, Occ: occipital bone, Pa: parietal bone, PMx: premaxillary bone.

To follow up on the consequences NC-loss has on mesenchymal specification and craniofacial development at later stages, we examined cartilage and bone development from E14.5 to E17.5. Due to embryonic lethality caused by homozygous *Pak1ip1* mutation no later stage mutants could be obtained. Our analysis revealed that while bone development appears delayed in *Pak1ip1*^*mray/mray*^ embryos, at E17.5 all bones of the skull formed correctly in the mutant and no other defects are apparent except for those immediately associated with midline clefting. Specifically, major cranial bones form with ∼24 h delay in homozygous *mray* mutants compared to WT and in accordance with the overall smaller habitus of mutant embryos are also smaller and discernably thinner (Figure 6). The shapes of both predominantly NC-derived, such as frontal bone, and mesodermal, such as parietal bone, of the mutant develop consistent with WT morphology. Notably, premaxillary and maxillary bones that are most closely associated with the orofacial cleft assume overall normal morphology. However, orofacial clefting remains prominently visible at later stages, as ventral views of skeletal stainings at E17.5 confirmed (Supplementary Figure S3). In summary, while cranial NCC appear severely diminished in *Pak1ip1*^*mray/mray*^ embryos during early developmental stages, gross dysmorphologies associated with this loss appear to be limited to the median cleft while NC-derived bones of the skull appear reduced in size in line with the overall size reduction of affected mutants.

## Discussion

Accumulating evidence suggests an overlap between ribosomopathies and neurocristopathies in association with a specific dependence of NCC development on proper ribosome biogenesis (Trainor and Merrill, 2014; Danilova and Gazda, 2015). The nature of this specific requirement remains elusive, but has been speculated to be centered on the uniquely rapid program of EMT that NCCs must undergo during their formation (Prakash et al., 2019). For EMT to occur, premigratory NCC that are located in a polarized epithelial layer, adjoined by adherens junctions and tight junctions, gradually lose their apical-basal cell polarity and dissolve their tight junctions. The accompanying cytoskeletal changes require a substantial change in the protein makeup of NCC to alter their adhesive properties so that they can dissociate from the neuroepithelium and emigrate (Lim and Thiery, 2012). Once adhesion has been sufficiently diminished, NCCs separate from surrounding cells and migrate extensively throughout the embryo directed to their target region, by a complex array of external signals (Mayor and Carmona-Fontaine, 2010; Theveneau and Mayor, 2012). Interestingly, this heightened vulnerability of NCCs to ribosome biogenesis dysfunction may also mediate the damaging effects of alcohol to developing NCCs and resulting craniofacial dysmorphology in fetal alcohol syndrome (Berres et al., 2017). Syndromes in which a clear association between defective ribosome biogenesis and NCC loss has been identified, include Treacher Collins syndrome (Dauwerse et al., 2011; Bowman et al., 2012), postaxial acrofacial dysostosis (Trainor and Merrill, 2014), acrofacial dysostosis – Cincinnati type (Weaver et al., 2015), Diamond Blackfan Anemia (Narla and Ebert, 2010), and Roberts syndrome (Xu et al., 2014).

Here, we report that mutation of the preribosomal factor Pak1ip1 in mice results in a specific loss of NCC early in development, following a cascade of Tp53/Ccng1 upregulation, G_0_/G_1_ cell cycle arrest, and cell death. The causal association between NCC loss and orofacial clefting is supported by the study of other genetic mouse models in which conditional inactivation of developmentally important genes, such as *Tgfbr2*, *Fgfr1*, and *Ptch1* in the developing NC leads to orofacial clefts of varying presentation (Ito et al., 2003; Metzis et al., 2013; Wang et al., 2013). Interestingly, in *Pak1ip1*^*mray/mray*^ mice NCC depletion is restricted to anterior aspects of the developing head and likely the principal cause of the observed midline cleft. This intriguing finding confirms that mutating factors that regulate the ubiquitously required process of ribosome biogenesis can nonetheless disproportionally affect one specific cell population, in this case, NCC of the frontonasal mesenchyme. The mechanisms by which some NCC are particularly dependent on Pak1ip1 activity remain elusive, but could be classified into two categories. First, Pak1ip1 is differentially expressed and its loss affects only cells within its expression domains. Second, subpopulations of NCC may have different requirements for survival and differentiation and only the most anterior population of NCC absolutely requires Pak1ip1. NCC populating the midfacial primordium may selectively produce specialized ribosomes, the biogenesis of which categorically requires Pak1ip1 function. In support of the former idea, RNA *in situ* hybridization in a previous study (Ross et al., 2013) demonstrated that *Pak1ip1* is nearly ubiquitously expressed, albeit with regional differences in expression levels. Notably, craniofacial structures extensively colonized by NCC, such as the facial prominences and branchial arches exhibited some of the highest *Pak1ip1* expression levels. The latter possibility is supported by observations of ribosomal diversity during mammalian development, which may distinguish otherwise similar cell populations with respect to their translational profiles and act as an additional layer of gene expression control (Kondrashov et al., 2011).

Despite commonalities with other mouse models of ribosomopathies (Nakhoul et al., 2014), *Pak1ip1*^*mray/mray*^ mice present with unique features clearly distinguishing them with respect to their craniofacial malformations. Strikingly, the midline cleft observed in affected *mray* mutant mice is a very rare dysmorphology found in mice and humans. Conditional inactivation of *Fgfr1* in NCC leads to midline clefting the encompassing maxillae and secondary palate (Wang et al., 2013). Intriguingly, a subset of *Tail short* (*Ts*) mice that carry causative mutations in the gene encoding ribosomal protein L38 (Rpl38) are characterized by a midfacial cleft phenotype identical to homozygous *mray* mutants (Kondrashov et al., 2011). In humans, median clefts are very uncommon appearing with a prevalence of 1 in 100,000 life births (Mishra et al., 2015). Of all cleft lip and palate cases, the incidence of median cleft lip has been placed between 0.17 and 0.73% (Apesos and Anigian, 1993; Dube and Jain, 2018), and considering the exceedingly small sample size no causative genes have been identified yet.

Craniofacial abnormalities in homozygous *Pak1ip1* mutants extend beyond the facial midline to include milder skull defects as well. While cartilage development appears intact in *Pak1ip1* mutants, bone growth is delayed and reaches comparable extent to the WT only by late gestation. Intriguingly, comparable deficits in bone development include both elements derived from NCC, but also elements derived from the mesoderm, such as parietal and occipital bones.

*Pak1ip1*^*mray/mray*^ embryos die prenatally, typically from E14.5 to E17.5. While the craniofacial abnormalities in mutant embryos are severe, they fail to explain this prenatal lethality, as other neurocristopathy models and craniofacial mouse models with comparable phenotypic alterations survive to term and even postnatal stages. Embryonic lethality caused by homozygous *Pak1ip* mutation may be best explained by the progressive loss of cells due to cell cycle arrest at G_0_/G_1_ phase, as comparative analysis of cell cycle progression suggests. Indeed, at E14.5 most cells in *Pak1ip1*^*mray/mray*^ embryos find themselves in G_0_/G_1_ phase, apparently a tipping point most embryos cannot overcome for development to progress further.

## Data Availability Statement

All datasets generated for this study are included in the article/Supplementary Material.

## Ethics Statement

The animal study was reviewed and approved by Association for Assessment and Accreditation of Laboratory Animal Care International (AALAC) and University of California at Davis Institutional Animal Care and Use Committee.

## Author Contributions

AP, AD, AL, AML, AR, and KZ conceived, conducted, and analyzed the experiments, performed the animal husbandry, and genotyping. KZ, AP, and AD wrote the manuscript. RM, PT, and LB provided experimental methods, tools, reagents, overall guidance, and co-wrote the manuscript. All authors contributed to the article and approved the submitted version.

## Conflict of Interest

The authors declare that the research was conducted in the absence of any commercial or financial relationships that could be construed as a potential conflict of interest.

## Supplementary Material

FIGURE S1 Correct patterning of the developing neural tube in *Pak1ip1*^*mray/mray*^ embryos. Pax3 immunofluorescent analysis comparing WT **(A)** to mutant **(B)** at E10.5 confirms proper Pax3 localization in the neural tube (NT) of *Pak1ip1*^*mray/mray*^ embryos. Scale bar is 100 μm.

FIGURE S2 Cell death in the head region of *Pak1ip1*^*mray/mray*^ embryos is substantially increased in late embryogenesis. Whole-mount embryos labeled *in vivo* during a 30 min interval with the acidic organelle probe LysoTracker. At both stages, E13.0 **(A,C)** and E14.5 **(B,D)**, substantial numbers of LysoTracker^+^ cells can be detected in the head region of the *Pak1ip1*^*mray/mray*^ embryos (asterisks), but not in the WT. Most LysoTracker^+^ cells are located in the epidermis, but also in the underlying mesenchyme.

FIGURE S3 Skeletal stainings in *Pak1ip1*^*mray/mray*^ embryos show overt orofacial clefting. Ventral views of the skull base stained with alcian blue and alizarin red of WT and mutant at E17.5 demonstrate orofacial clefting affecting premaxillary (PMx) and palatine bones (Pa) (asterisk). The palatal shelves of the *Pak1ip1*^*mray/mray*^ embryo failed to grow towards the midline revealing the overlying presphenoid bone (PS). BO: basioccipital bone, BS: basisphenoid bone, Mx: maxillary bone.

## References

[B1] Apesos J. Anigian G. M. (1993). Median cleft of the lip: its significance and surgical repair. *Cleft. Palate Craniofac. J.* 30 94–96. 10.1597/1545-1569_1993_030_0094_mcotli_2.3.co_2 8418880

[B2] Berres M. E. Garic A. Flentke G. R. Smith S. M. (2017). Transcriptome profiling identifies ribosome biogenesis as a target of alcohol teratogenicity and vulnerability during early embryogenesis. *PLoS One* 12:e0169351. 10.1371/journal.pone.0169351 28046103 PMC5207668

[B3] Bowman M. Oldridge M. Archer C. O’Rourke A. McParland J. Brekelmans R. (2012). Gross deletions in TCOF1 are a cause of Treacher-Collins-Franceschetti syndrome. *Eur. J. Hum. Genet.* 20 769–777. 10.1038/ejhg.2012.2 22317976 PMC3376267

[B4] Cordero D. R. Brugmann S. Chu Y. Bajpai R. Jame M. Helms J. A. (2011). Cranial neural crest cells on the move: their roles in craniofacial development. *Am. J. Med. Genet. A* 155 270–279. 10.1002/ajmg.a.33702 21271641 PMC3039913

[B5] Danielian P. S. Muccino D. Rowitch D. H. Michael S. K. McMahon A. P. (1998). Modification of gene activity in mouse embryos in utero by a tamoxifen-inducible form of Cre recombinase. *Curr. Biol.* 8 1323–1326. 10.1016/s0960-9822(07)00562-3 9843687

[B6] Danilova N. Gazda H. T. (2015). Ribosomopathies: how a common root can cause a tree of pathologies. *Dis. Model Mech.* 8 1013–1026. 10.1242/dmm.020529 26398160 PMC4582105

[B7] Dauwerse J. G. Dixon J. Seland S. Ruivenkamp C. A. van Haeringen A. Hoefsloot L. H. (2011). Mutations in genes encoding subunits of RNA polymerases I and III cause treacher collins syndrome. *Nat. Genet.* 43 20–22. 10.1038/ng.724 21131976

[B8] Draptchinskaia N. Gustavsson P. Andersson B. Pettersson M. Willig T. N. Dianzani I. (1999). The gene encoding ribosomal protein S19 is mutated in Diamond-Blackfan anaemia. *Nat. Genet.* 21 169–175. 10.1038/5951 9988267

[B9] Dube G. Jain S. (2018). Prevalence of true median cleft of upper lip as reflected from a small central indian population: attempt to report and review the true median cleft of upper lip. *J. Maxillofac. Oral. Surg.* 17 508–513. 10.1007/s12663-017-1072-1 30344394 PMC6181860

[B10] Fogel J. L. Thein T. Z. Mariani F. V. (2012). Use of LysoTracker to detect programmed cell death in embryos and differentiating embryonic stem cells. *J. Vis. Exp.* 11:4254. 10.3791/4254 PMC3490301 23092960

[B11] Gammill L. S. Gonzalez C. Bronner-Fraser M. (2007). Neuropilin 2/semaphorin 3F signaling is essential for cranial neural crest migration and trigeminal ganglion condensation. *Dev. Neurobiol.* 67 47–56. 10.1002/dneu.20326 17443771

[B12] Gazda H. T. Grabowska A. Merida-Long L. B. Latawiec E. Schneider H. E. Lipton J. M. (2006). Ribosomal protein S24 gene is mutated in Diamond-Blackfan anemia. *Am. J. Hum. Genet.* 79 1110–1118. 17186470 10.1086/510020 PMC1698708

[B13] Helms J. A. Schneider R. A. (2003). Cranial skeletal biology. *Nature* 423 326–331. 10.1038/nature01656 12748650

[B14] Ito Y. Yeo J. Y. Chytil A. Han J. Bringas P. Nakajima A. Jr. (2003). Conditional inactivation of Tgfbr2 in cranial neural crest causes cleft palate and calvaria defects. *Development* 130 5269–5280. 10.1242/dev.00708 12975342

[B15] Kondrashov N. Pusic A. Stumpf C. R. Shimizu K. Hsieh A. C. Xue S. (2011). Ribosome-mediated specificity in Hox mRNA translation and vertebrate tissue patterning. *Cell* 145 383–397. 10.1016/j.cell.2011.03.028 21529712 PMC4445650

[B16] Lim J. Thiery J. P. (2012). Epithelial-mesenchymal transitions: insights from development. *Development* 139 3471–3486. 10.1242/dev.071209 22949611

[B17] Liu J. Xu Y. Stoleru D. Salic A. (2012). Imaging protein synthesis in cells and tissues with an alkyne analog of puromycin. *Proc. Natl. Acad. Sci. U.S.A.* 109 413–418. 10.1073/pnas.1111561108 22160674 PMC3258597

[B18] Mayor R. Carmona-Fontaine C. (2010). Keeping in touch with contact inhibition of locomotion. *Trends Cell Biol.* 20 319–328. 10.1016/j.tcb.2010.03.005 20399659 PMC2927909

[B19] Metzis V. Courtney A. D. Kerr M. C. Ferguson C. Rondon Galeano M. C. Parton R. G. (2013). Patched1 is required in neural crest cells for the prevention of orofacial clefts. *Hum. Mol. Genet.* 22 5026–5035. 10.1093/hmg/ddt353 23900075

[B20] Minoux M. Rijli F. M. (2010). Molecular mechanisms of cranial neural crest cell migration and patterning in craniofacial development. *Development* 137 2605–2621. 10.1242/dev.040048 20663816

[B21] Mishra S. Sabhlok S. Panda P. K. Khatri I. (2015). Management of midline facial clefts. *J. Maxillofac. Oral. Surg.* 14 883–890. 10.1007/s12663-015-0763-8 26604459 PMC4648772

[B22] Nakhoul H. Ke J. Zhou X. Liao W. Zeng S. X. Lu H. (2014). Ribosomopathies: mechanisms of disease. *Clin. Med. Insights Blood Disord.* 7 7–16. 25512719 10.4137/CMBD.S16952 PMC4251057

[B23] Narla A. Ebert B. L. (2010). Ribosomopathies: human disorders of ribosome dysfunction. *Blood* 115 3196–3205. 10.1182/blood-2009-10-178129 20194897 PMC2858486

[B24] Nazar R. N. (2004). Ribosomal RNA processing and ribosome biogenesis in eukaryotes. *IUBMB Life* 56 457–465. 10.1080/15216540400010867 15545225

[B25] Okamoto K. Beach D. (1994). Cyclin G is a transcriptional target of the p53 tumor suppressor protein. *EMBO J.* 13 4816–4822. 10.1002/j.1460-2075.1994.tb06807.x 7957050 PMC395420

[B26] Prakash V. Carson B. B. Feenstra J. M. Dass R. A. Sekyrova P. Hoshino A. (2019). Ribosome biogenesis during cell cycle arrest fuels EMT in development and disease. *Nat. Commun.* 10:2110. 10.1038/s41467-019-10100-8 PMC6506521 31068593

[B27] Ross A. P. Mansilla M. A. Choe Y. Helminski S. Sturm R. Maute R. L. (2013). A mutation in mouse pak1ip1 causes orofacial clefting while human PAK1IP1 Maps to 6p24 translocation breaking points associated with orofacial clefting. *PLoS One* 8:e69333. 10.1371/journal.pone.0069333 23935987 PMC3723895

[B28] Saveanu C. Rousselle J. C. Lenormand P. Namane A. Jacquier A. Fromont-Racine M. (2007). The p21-activated protein kinase inhibitor Skb15 and its budding yeast homologue are 60S ribosome assembly factors. *Mol. Cell Biol.* 27 2897–2909. 10.1128/mcb.00064-07 17308036 PMC1899936

[B29] Schwarz Q. Vieira J. M. Howard B. Eickholt B. J. Ruhrberg C. (2008). Neuropilin 1 and 2 control cranial gangliogenesis and axon guidance through neural crest cells. *Development* 135 1605–1613. 10.1242/dev.015412 18356247 PMC2705499

[B30] Soo K. O’Rourke M. P. Khoo P. L. Steiner K. A. Wong N. Behringer R. R. (2002). Twist function is required for the morphogenesis of the cephalic neural tube and the differentiation of the cranial neural crest cells in the mouse embryo. *Dev. Biol.* 247 251–270. 10.1006/dbio.2002.0699 12086465

[B31] Teber O. A. Gillessen-Kaesbach G. Fischer S. Bohringer S. Albrecht B. Albert A. (2004). Genotyping in 46 patients with tentative diagnosis of Treacher Collins syndrome revealed unexpected phenotypic variation. *Eur. J. Hum. Genet.* 12 879–890. 10.1038/sj.ejhg.5201260 15340364

[B32] Theveneau E. Mayor R. (2012). Neural crest delamination and migration: from epithelium-to-mesenchyme transition to collective cell migration. *Dev. Biol.* 366 34–54. 10.1016/j.ydbio.2011.12.041 22261150

[B33] Trainor P. A. (2010). Craniofacial birth defects: the role of neural crest cells in the etiology and pathogenesis of treacher collins syndrome and the potential for prevention. *Am. J. Med. Genet. A* 152 2984–2994. 10.1002/ajmg.a.33454 20734335 PMC3686507

[B34] Trainor P. A. Merrill A. E. (2014). Ribosome biogenesis in skeletal development and the pathogenesis of skeletal disorders. *Biochim. Biophys. Acta* 1842 769–778. 10.1016/j.bbadis.2013.11.010 24252615 PMC4020712

[B35] Wang C. Chang J. Y. Yang C. Huang Y. Liu J. You P. (2013). Type 1 fibroblast growth factor receptor in cranial neural crest cell-derived mesenchyme is required for palatogenesis. *J. Biol. Chem.* 288 22174–22183. 10.1074/jbc.m113.463620 23754280 PMC3724669

[B36] Weaver K. N. Watt K. E. Hufnagel R. B. Navajas Acedo J. Linscott L. L. Sund K. L. (2015). Acrofacial dysostosis, cincinnati type, a mandibulofacial dysostosis syndrome with limb anomalies, is caused by POLR1A Dysfunction. *Am. J. Hum. Genet.* 96 765–774. 10.1016/j.ajhg.2015.03.011 25913037 PMC4570288

[B37] Xu B. Lu S. Gerton J. L. (2014). Roberts syndrome: a deficit in acetylated cohesin leads to nucleolar dysfunction. *Rare Dis.* 2:e27743. 10.4161/rdis.27743 25054091 PMC4091327

[B38] Yu W. Qiu Z. Gao N. Wang L. Cui H. Qian Y. (2011). PAK1IP1, a ribosomal stress-induced nucleolar protein, regulates cell proliferation via the p53-MDM2 loop. *Nucleic Acids Res.* 39 2234–2248. 10.1093/nar/gkq1117 21097889 PMC3064775

[B39] Zarbalis K. Wurst W. (2000). Expression domains of murine ephrin-A5 in the pituitary and hypothalamus. *Mech. Dev.* 93 165–168. 10.1016/s0925-4773(00)00252-5 10781950

[B40] Zhao L. Samuels T. Winckler S. Korgaonkar C. Tompkins V. Horne M. C. (2003). Cyclin G1 has growth inhibitory activity linked to the ARF-Mdm2-p53 and pRb tumor suppressor pathways. *Mol. Cancer Res.* 1 195–206. 12556559

