# Supplementary figures and images for: Pak1ip1 Loss-of-Function Leads to Cell Cycle Arrest, Loss of Neural Crest Cells, and Craniofacial Abnormalities
Source: Front Cell Dev Biol. 2020 Sep 1;8:510063. doi: 10.3389/fcell.2020.510063 (PMC7490522; doi:10.3389/fcell.2020.510063)

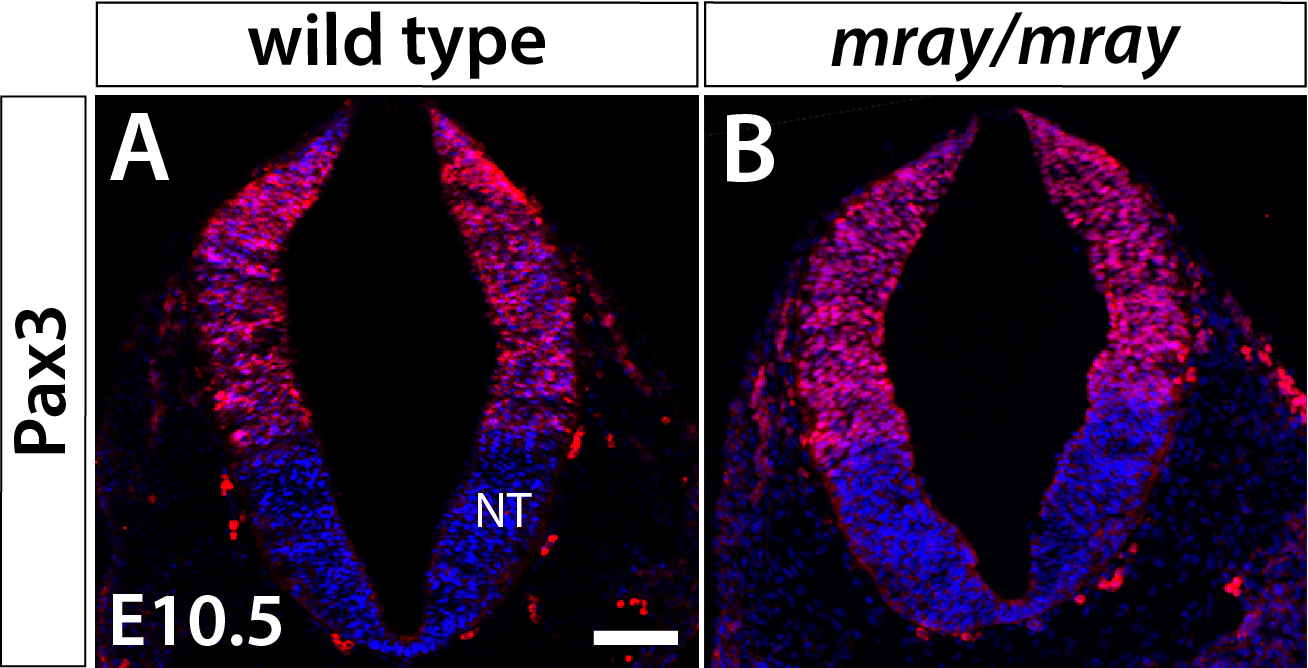

Supplement: FIGURE S1 — Correct patterning of the developing neural tube in Pak1ip1mray/mray embryos. Pax3 immunofluorescent analysis comparing WT (A) to mutant (B) at E10.5 confirms proper Pax3 localization in the neural tube (NT) of Pak1ip1mray/mray embryos. Scale bar is 100 μm. [file Image_1.TIF]

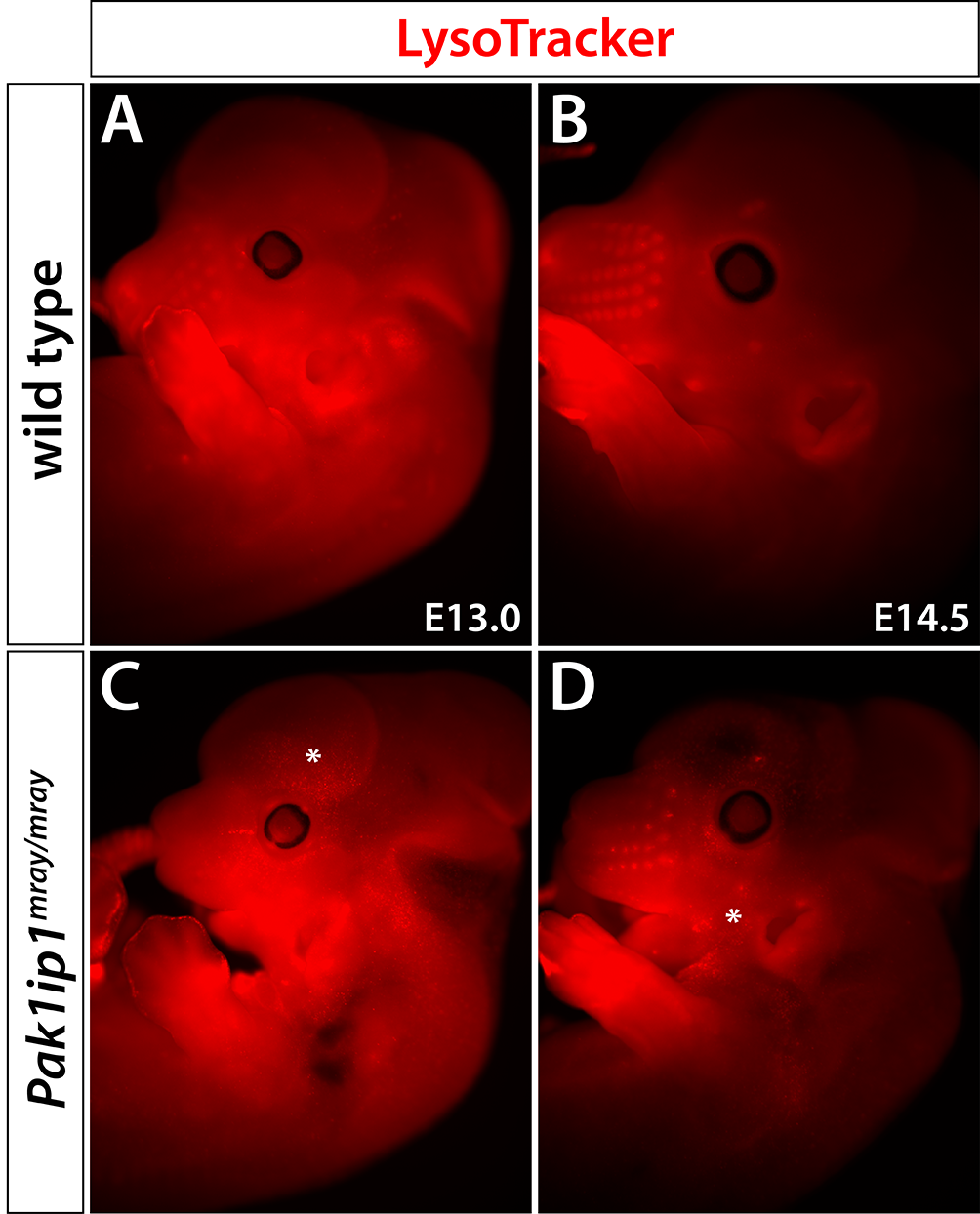

Supplement: FIGURE S2 — Cell death in the head region of Pak1ip1mray/mray embryos is substantially increased in late embryogenesis. Whole-mount embryos labeled in vivo during a 30 min interval with the acidic organelle probe LysoTracker. At both stages, E13.0 (A,C) and E14.5 (B,D), substantial numbers of LysoTracker+ cells can be detected in the head region of the Pak1ip1mray/mray embryos (asterisks), but not in the WT. Most LysoTracker+ cells are located in the epidermis, but also in the underlying mesenchyme. [file Image_2.TIF]

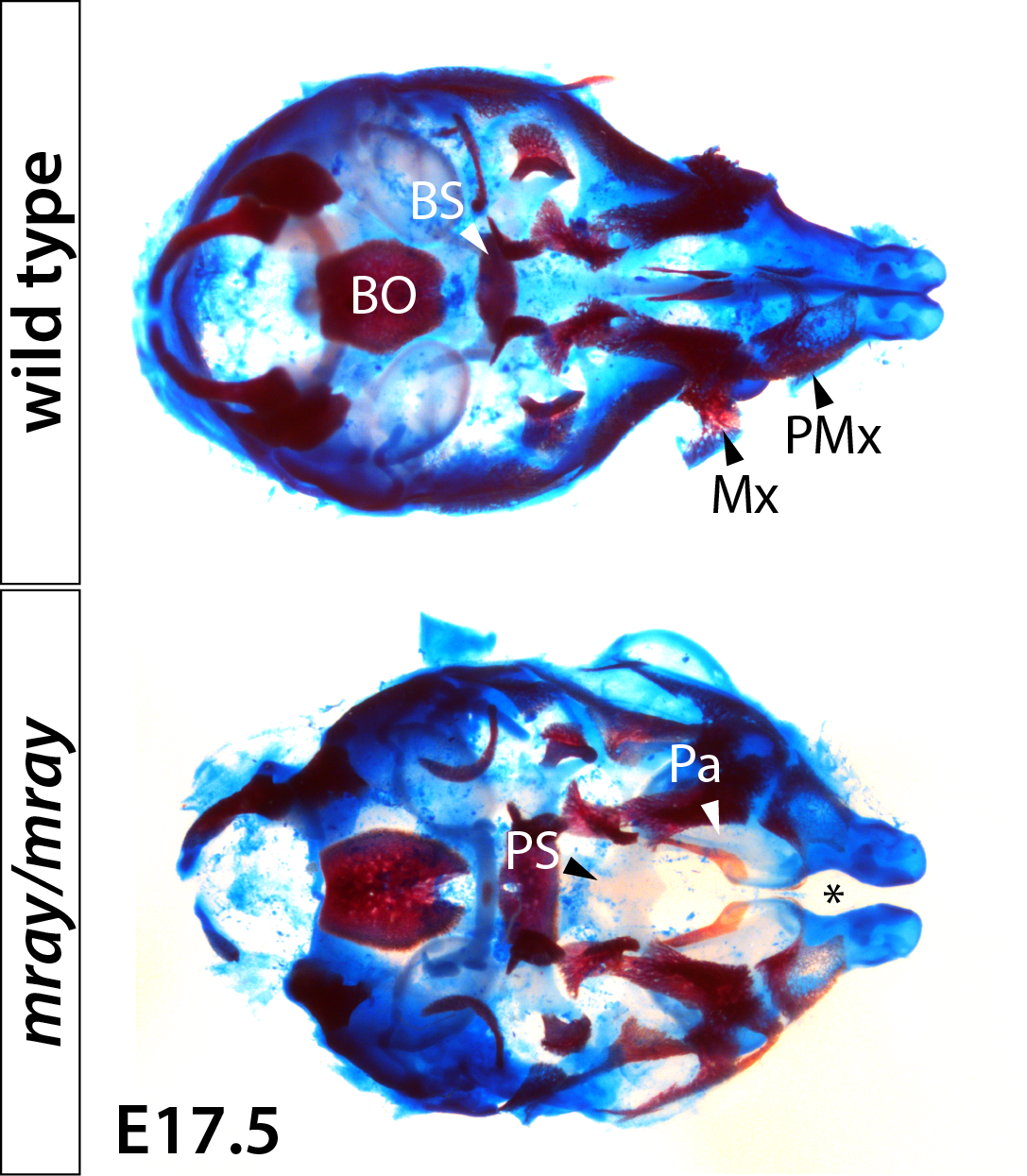

Supplement: FIGURE S3 — Skeletal stainings in Pak1ip1mray/mray embryos show overt orofacial clefting. Ventral views of the skull base stained with alcian blue and alizarin red of WT and mutant at E17.5 demonstrate orofacial clefting affecting premaxillary (PMx) and palatine bones (Pa) (asterisk). The palatal shelves of the Pak1ip1mray/mray embryo failed to grow towards the midline revealing the overlying presphenoid bone (PS). BO: basioccipital bone, BS: basisphenoid bone, Mx: maxillary bone. [file Image_3.TIF]
